# Imported Methicillin-Resistant *Staphylococcus aureus*, Sweden

**DOI:** 10.3201/eid1602.081655

**Published:** 2010-02

**Authors:** Mikael Stenhem, Åke Örtqvist, Håkan Ringberg, Leif Larsson, Barbro Olsson-Liljequist, Sara Hæggman, Mats Kalin, Karl Ekdahl

**Affiliations:** Swedish Institute for Infectious Disease Control, Solna, Sweden (M. Stenhem, B. Olsson-Liljequist, S. Hæggman); Karolinska Institutet, Solna (M. Stenhem, Å. Örtqvist, M. Kalin, K. Ekdahl); County Medical Office Communicable Disease Control, Stockholm, Sweden (Å. Örtqvist); Regional Centre for Communicable Disease Control and Prevention, Malmö, Sweden (H. Ringberg); Sahlgrenska University Hospital, Göteborg, Sweden (L. Larsson); European Centre for Disease Prevention and Control, Solna (K. Ekdahl)

**Keywords:** Communicable disease control, community-acquired infections, disease transmission, antimicrobial resistance, molecular epidemiology, methicillin-resistant Staphylococcus aureus, staphylococci, population surveillance, Sweden, research

## Abstract

Countries such as Sweden that have a low prevalence of methicillin-resistant *Staphylococcus aureus* (MRSA) offer the opportunity to discern and study transmission of imported cases of MRSA. We analyzed 444 imported cases of MRSA acquisition reported in Sweden during 2000–2003. Risk for MRSA in returning travelers ranged from 0.1 (95% confidence interval [CI] 0.01–0.4) per 1 million travelers to Nordic countries to 59.4 (95% CI 44.5–79.3) per 1 million travelers to North Africa and the Middle East. Most imported cases (246, 55%) were healthcare acquired, but regions with the highest risk for MRSA in travelers showed a correlation with community acquisition (r = 0.81, p = 0.001). Characteristic differences in MRSA strains acquired were dependent on the region from which they originated and whether they were community or healthcare acquired. Knowledge of differences in transmission of MRSA may improve control measures against imported cases.

## Abstract 2

Knowledge of different risks for infection will improve control measures.

Global transmission of methicillin-resistant *Staphylococcus aureus* (MRSA) has been the subject of many studies. Conclusions have commonly been drawn from the occurrence of identical or similar strains in different countries or regions (*1*–*6*). Studies of individual MRSA cases linked to international travel have often been in the form of case reports, case series, or other descriptive reports (*7*–*11*). Only a few small analytical or population-based studies have been published (*12*–*14*). In countries with a high prevalence of MRSA, imported cases of MRSA are not easily distinguished from the domestic background prevalence. Thus, low prevalence countries, such as Sweden, offer a better opportunity to discern and study imported cases of MRSA.

The primary purpose of our study was to use validated data from the surveillance system in Sweden to analyze the risk for MRSA among Swedish residents traveling abroad, internationally adopted children, and immigrants. A secondary purpose was to determine whether different types of MRSA were acquired in different countries or regions.

## Materials and Methods

### Data on MRSA Cases in the Statutory Swedish Communicable Disease Notification System

The study was reviewed and approved by the Regional Ethics Approval Board in Stockholm (Protocol 2005/4:10). MRSA cases (infections as well as carriage) are notified in parallel to the Swedish Institute for Infectious Disease Control (SMI) by the doctor caring for the patient and by the bacteriological laboratory having isolated the MRSA strain, using a unique personal identification number issued to all Swedish residents and used in all contacts with the health care system. At SMI, a case record is created after the arrival of the first notification. Any subsequent notifications are merged with the initial case record. After completion of the epidemiologic investigation and contact tracing around each MRSA case, the epidemiologic notification form data are validated by SMI with input from MRSA contact persons, who are public health representatives involved in control efforts locally in the respective counties (*15*). The validated variables used in the study were age, sex, transmission setting (healthcare acquired [HA] or community acquired [CA]), country of acquisition, and purpose of travel (leisure or work related) (*16*). If a person infected with MRSA had been abroad <6 months before MRSA was diagnosed and either had a strain that was present in other persons from that region or had one that was previously unknown in Sweden and no likely source of MRSA in Sweden was found in the epidemiologic investigation, that person was considered to have acquired MRSA abroad. If there was a reasonable possibility that MRSA could have been acquired either domestically or abroad, country of acquisition was considered unknown. If the person infected with MRSA had been admitted to or had worked in a healthcare setting during his or her stay abroad, he or she was considered to have a case of HA MRSA. Information in the study database on whether a person had MRSA disease or MRSA carriage was based on clinical case data from the physician caring for the patient.

For additional analysis, the reported cases of MRSA were divided into the following categories: residents of Sweden traveling abroad for recreational purposes, residents of Sweden traveling abroad because of work or studies, foreign residents traveling to Sweden for a temporary visit, immigrants, internationally adopted children, or Swedish citizens returning home after having lived abroad for >1 year. The risk for MRSA in travelers, immigrants, and adopted children was also evaluated by including and excluding reported cases found through public health-initiated case-finding activities and secondary cases in transmission chains.

### Swedish Tourist and Travel Database

Travel data from the commercial Swedish Tourist and Travel Database (TDB) were used as a denominator when analyzing the risk for being reported as having MRSA in travelers from Sweden. The TDB is based on monthly interviews with 2,000 randomly selected residents of Sweden. We used the TDB variables age group, sex, purpose of travel (leisure or work), main destination, and time of travel. TDB for 2000–2003 and interviews of 9,213 persons with a history of travel abroad were used. Information in the TDB was weighted and extrapolated to estimate the total number of travelers from Sweden and to derive a total of 45,855,000 travel episodes (*17*). Actual interviewees were used as controls in an adjusted model that estimated the odds ratio (OR) of having MRSA, and extrapolated estimates were used to calculate risks per 1,000,000 travelers. Because only persons <75 years of age were sampled for interviews for the TDB, 34 cases of MRSA in persons >74 years of age were excluded from the TDB analyses.

### Data on Immigration and International Adoptions

Denominator data on the yearly number of immigrants to Sweden were obtained from Statistics Sweden (*18*). Included in the immigration figures were persons who received a residence permit because of family ties, European Economic Area convention, and labor permits; visiting students; and asylum seekers per country of citizenship. Data on the yearly number of international adoptions per country were obtained from the Swedish Intercountry Adoptions Authority (*19*).

### Epidemiologic Typing of MRSA Isolates

Isolates from all persons with new cases of MRSA were sent to SMI, where laboratory diagnosis of MRSA was confirmed by using PCR for the *mecA* and the *nuc* genes (*20*). Epidemiologic typing of all isolates was performed by using pulsed-field gel electrophoresis (PFGE) according to the protocol of Murchan et al. (*21*). On the basis of PFGE results, representative isolates were selected for multilocus sequence typing (MLST) (*22*), and sequence types (STs) of isolates were determined by using the MLST database (www.mlst.net).

### Statistical Analysis

Stata 8.2 software (StataCorp LP, College Station, TX, USA) was used for all statistical analyses, and a 95% level of significance was applied. Sex, age group, purpose of travel, and destination were used as explanatory variables in multivariable analysis of travelers with a diagnosis of MRSA and its reporting as an outcome. Generalized estimating equations with independent correlation and robust standard errors were used to enable clustering of data in the adjusted model that included all travelers <75 years of age.

## Results

During 2000–2003, a total of 1,733 cases of MRSA were reported in Sweden, 444 of which were considered to have been acquired abroad. Among these imported cases, 292 (65.8%) were in residents of Sweden who traveled abroad; 31 (7.0%) had traveled for work reasons or studies. Fifty-six (12.6%) of the cases were in newly arrived immigrants to Sweden, 40 (9.0%) in internationally adopted children from non-Nordic countries, 30 (6.8%) in foreign residents visiting Sweden, 20 (4.5%) in citizens of Sweden returning home after having lived abroad for >1 year, and 6 (1.4%) in persons who could not be grouped. Of the imported MRSA cases, 246 (55.4%) were HA and 146 (32.9%) were CA; 190 (42.8%) were in persons with MRSA disease.

### MRSA in Travelers Returning to Sweden

Of the 292 residents of Sweden who traveled abroad, 267 met the age criteria and were included in this analysis. Most persons with MRSA had traveled to Thailand (n = 33), the United Kingdom (n = 24), Greece (n = 22), the former Yugoslavia (n = 20), Lebanon (n = 16), the United States (n = 14), Cyprus (n = 12), Spain (n = 12), Turkey (n = 9), and Syria (n = 8). The overall risk for being reported as having MRSA was 5.8 cases/1,000,000 travelers (95% confidence interval [CI] 5.2–6.6). The crude risk for travelers to specific regions ranged from 0.1 (95% CI 0.01–0. 4) per 1,000,000 travelers to Nordic countries to 59.4 (95% CI 44.5–79.3) per 1,000,000 travelers to North Africa and the Middle East (Table 1). Rank in risk for MRSA in the regions analyzed changed only slightly when we adjusted for confounders.

**Table 1:** Estimated number of travelers and cases of travel-associated acquisition of MRSA, Sweden, 2000–2003*

Variable	Estimated no. travelers	No. with MRSA infection (no. with MRSA disease)	Crude risk/1 million travelers (95% CI)	% Community-acquired cases	Adjusted OR (95% CI)
Sex					
F	25,219,000	109 (62)	4.3 (3.6–5.2)	42.2	Reference
M	20,634,000	158 (71)	7.7 (6.6–8.9)	27.2	1.5 (1.2–2.0)
Age group, y					
0–14	6,497,000	30 (24)	4.6 (3.2–6.6)	83.3	Reference
15–29	8,657,000	72 (36)	8.3 (6.6–10)	31.9	1.0 (0.6–1.5)
30–44	13,234,000	49 (26)	3.7 (2.8–4.9)	44.9	0.8 (0.5–1.3)
45–59	12,342,000	62 (29)	5.0 (3.9–6.4)	22.6	0.8 (0.5–1.2)
60–74	5,127,000	54 (18)	11 (8.1–13.8)	9.3	1.2 (0.7–1.9)
Purpose of travel					
Leisure	36,323,000	237 (123)	6.5 (5.7–7.4)	34.2	Reference
Work	9,532,000	30 (10)	3.1 (2.2–4.5)	26.7	2.4 (1.5–3.8)
Region of origin†					
Nordic	15,962,000	1 (0)	0.1 (0.01–0.4)	0.0	0.1 (0.01–0.6)
Western Europe	7,614,000	7 (3)	0.9 (0.4–1.9)	14.3	Reference
Southern Europe	7,601,000	18 (5)	2.4 (1.5–3.8)	0.0	2.4 (1.0–5.8)
Central and Eastern Europe	2,885,000	7 (5)	2.4 (1.2–5.1)	28.6	2.8 (1.0–8.1)
UK and Ireland	2,815,000	26 (11)	9.2 (6.3–13.6)	7.7	10.3 (4.4–24..0)
North America	1,627,000	17 (4)	10.4 (6.5–16.8)	35.3	10.6 (4.2–26.7)
Northeastern Mediterranean	4,317,000	63 (30)	14.6 (11.4–18.7)	38.1	15.8 (7.0–35.6)
South America	152,000	6 (5)	39.5 (17.7–87.9)	33.3	31.2 (10.0–97.6)
East Asia	1,622,000	54 (27)	33.3 (25.5–43.5)	27.8	36.5 (16.2–82.0)
Oceania and Pacific Islands	250,000	11 (5)	44.0 (24.4–79.5)	36.4	43.0 (15.5–119.4)
Sub-Saharan Africa	232,000	11 (7)	47.4 (26.3–85.6)	54.5	46.3 (17.3–123.6)
North Africa and Middle East	774,000	46 (31)	59.4 (44.5–79.3)	58.7	59.0 (25.1–138.9)
Total	45,855,000	267 (133)	5.8 (5.2–6.6)	33.3	

*MRSA, methicillin-resistant *Staphylococcus aureus*; CI, confidence interval; OR, odds ratio. †Nordic: Denmark, Finland, Iceland, Norway; Western Europe: Austria, Belgium, France, Germany, Luxembourg, the Netherlands, Switzerland; Southern Europe: Italy, Malta, Monaco, Portugal, Spain; Central and Eastern Europe: Belarus, Bulgaria, Estonia, Hungary, Kazakhstan, Latvia, Lithuania, Poland, Romania, Russia, Slovakia, Czech Republic, Ukraine; North America: Canada, Cuba, Dominican Republic, Guadeloupe, Guatemala, Mexico, Panama, United States; Northeastern Mediterranean: Albania, Bosnia-Herzegovina, Cyprus, former Yugoslavia, Greece, Croatia, Macedonia, Serbia, Slovenia, Turkey; South America: Brazil, Chile, Colombia, Ecuador, Peru, Venezuela; East Asia: Afghanistan, Bangladesh, Cambodia, China, Hong Kong, India, Indonesia, Japan, South Korea, Malaysia, Maldives, Mongolia, Pakistan, Singapore, Sri Lanka, Taiwan, Thailand, Vietnam, the Philippines; Oceania and Pacific islands: Australia, Hawaii, New Zealand, Samoa; Sub-Saharan Africa: Angola, Botswana, Chad, Ethiopia, the Gambia, Ghana, Kenya, Congo–Kinshasa, Madagascar, Senegal, Sierra Leone–Togo, Somalia, Sudan, Swaziland, South Africa, Tanzania–Uganda; North Africa and Middle East: Algeria, Egypt, Iraq, Iran, Israel, Jordan, Kuwait, Lebanon, Libya, Morocco, Saudi Arabia, Syria, Tunisia, United Arab Emirates.

The adjusted OR for having MRSA was 1.5× higher among male participants (95% CI 1.2–2.0) than female participants (Table 1). The crude risk differed slightly between age groups and was highest among persons 60–74 years of age (11 cases/1,000,000 travelers; 95% CI 8.1–13.8), but no difference was found in the OR between age groups in the adjusted model. Work-related travel showed a lower crude risk for being reported as having MRSA, but when adjusting for other risk factors, the OR of MRSA was 2.4× higher among persons who traveled for work purposes than in leisure travelers (95% CI 1.5–3.8). To examine to what degree inclusion or exclusion of carriers would change our findings, analysis was repeated by using only the 133 (49.8%) cases in travelers with MRSA disease. We found only minor changes in ranking of ORs for geographic regions. The same 5 regions (Table 1) still ranked highest with higher ORs than other regions. The most prominent difference for ORs was that South America and Oceania and Pacific Islands changed places in rank.

Percentages of CA-MRSA for the respective regions, correlated linearly with adjusted ORs (r = 0.81, p = 0.001). Among age groups, the proportion of cases of CA-MRSA was highest in persons 0–14 years of age (83.3%), lowest in persons 60–74 years of age (9.3%), and intermediate for other age groups.

### MRSA in Immigrants

A total of 56 persons who had recently arrived in Sweden as immigrants had cases of MRSA (Table 2), giving an overall risk of 15.9 (95% CI 12.0– 20.7) cases/100,000 immigrants. MRSA disease was present in 27 (48.2%) of the immigrants. Twenty of the persons with MRSA came from the former Yugoslavia. The highest number of immigrants (n = 45,587) also came from this country; these persons had a risk of 43.8 (95% CI 26.8–67.8) MRSA cases/100,000 immigrants. Nearly as many immigrants came from Iraq, but the risk for MRSA was lower for this group (9.1, 95% CI 2.5–23.4 cases/100,000 immigrants).

**Table 2:** Imported cases of MRSA acquisition and disease among immigrants, Sweden, 2000–2003*

Country of origin	No. immigrants†	No. with MRSA acquisition (no. with MRSA disease)	Risk/100,000 immigrants (95% CI)
Malta	21	1 (0)	4,761.9 (120.5–23,816.0)
Kazakhstan	933	4 (2)	428.7 (116.9–1,094.0)
South Africa	556	2 (1)	359.7 (43.6–1,293.3)
Sudan	360	1 (1)	277.8 (7.0–1,537.9)
Italy	1,382	2 (1)	144.7 (17.5–521.8)
Ecuador	995	1 (1)	100.5 (2.5–558.6)
Lebanon	2,100	2 (0)	95.2 (11.5–343.6)
Mongolia	1,055	1 (1)	94.8 (2.4–527.0)
Japan	1,274	1 (0)	78.5 (2.0–436.6)
The Philippines	1,335	1 (1)	74.9 (1.9–416.6)
Afghanistan	6,166	4 (3)	64.9 (17.7–166.0)
United States	4,094	2 (0)	48.9 (5.9–176.4)
Former Yugoslavia	45,587	20 (10)	43.8 (26.8–67.8)
Germany	6,564	2 (0)	30.5 (3.7–110.0)
Poland	3,658	1 (0)	27.3 (0.7–152.2)
Iran	7,856	2 (1)	25.5 (3.1–91.9)
Thailand	4,883	1 (0)	20.5 (0.5–114.0)
United Kingdom	5,306	1 (0)	18.9 (0.47.7–105.0)
Turkey	5,568	1 (1)	18.0 (0.5–100.0)
Russia	8,254	1 (0)	12.1 (0.3–67.5)
Somalia	8,546	1 (1)	11.7 (0.3–65.2)
Iraq	43,730	4 (3)	9.1 (2.5–23.4)
Other countries	191,302	0	–
Total	351,525	56 (27)	15.9 (12.0–20.7)

*MRSA, methicillin-resistant *Staphylococcus aureus*; CI, confidence interval. †Included in the immigration figures were persons who received a residence permit because of family ties, European Economic Area convention, and labor permits; visiting students; and asylum seekers per country of citizenship.

### MRSA in Adopted Children

In 2000–2003, a total of 4,169 children were adopted from non-Nordic countries. Forty adopted children from 12 countries were reported as having cases of MRSA shortly after their arrival in Sweden, i.e., 9.6 (95% CI 6.9–13.0) cases per 1,000 adoptions (Table 3). Of these adopted children with MRSA, 6 had MRSA disease. Risk for having MRSA ranged from 3.0/1,000 adoptions of children from Vietnam (95% CI 0.1–16.8) to 74.1/1,000 adoptions of children from the Philippines (95% CI 9.1–242.8). The largest number of adopted children (n = 1,074) came from the People’s Republic of China. However, only 6.5/1,000 adoptees (95% CI 2.6–13.4) from this country had MRSA. Children adopted from South Korea showed 30.0 cases/1,000 adoptions (95% CI 16.5–49.8).

**Table 3:** Imported cases of MRSA acquisition and disease among children adopted from other countries, Sweden, 2000–2003*

Country	No. adoptions	No. with MRSA acquisition (no. with MRSA disease)	Risk/1,000 adoptions (95% CI)
The Philippines	27	2 (0)	74.1 (9.1–242.8)
South Korea	467	14 (0)	30.0 (16.5–49.8)
Bulgaria	91	2 (1)	22.0 (2.7–77.1)
Belarus	174	3 (1)	17.2 (3.6–49.6)
Russia	263	4 (0)	15.2 (4.2–38.5)
Ethiopia	71	1 (1)	14.1 (0.4–76.0)
Ukraine	74	1 (0)	13.5 (0.3–73.0)
People’s Republic of China	1,074	7 (3)	6.5 (2.6–13.4)
Colombia	460	3 (0)	6.5 (1.3–18.9)
South Africa	154	1 (0)	6.5 (0.16–35.6)
India	243	1 (0)	4.1 (0.1–22.7)
Vietnam	328	1 (0)	3.0 (0.1–16.8)
Other countries	743	0	–
Total	4,169	40 (6)	9.6 (6.9–13.0)

*MRSA, methicillin-resistant *Staphylococcus aureus*; CI, confidence interval.

### Type of MRSA and Geographic Region of Acquisition

STs inferred from PFGE results and region of acquisition for 414 MRSA cases (excluding secondary cases and cases with STs seen in <5 cases) are shown in the Appendix Table. Also shown are the number of HA-MRSA and CA-MRSA cases per ST and region of acquisition. HA ST22 and ST36 predominated in cases from the United Kingdom and Ireland (15/33 and 11/33 MRSA cases, respectively). Among 92 cases from East Asia, the predominant MRSA were HA ST239 (n = 22 cases), CA ST30 (n = 13 cases), and CA ST72 (n = 6 cases). CA ST30 isolates were also seen in 5 cases from neighboring areas (3 from the Oceania and Pacific Islands region and 2 from Hawaii that belonged to North American region). Among the 50 cases from North Africa and the Middle East, CA ST80 (n = 18 cases) dominated. ST 80 was also isolated from 21 of 90 cases from the neighboring northeastern Mediterranean region. However, this region was mainly characterized by 30 HA ST239 (n = 30 cases). In cases from southern Europe, HA ST228 was most frequently seen (11/36 cases). Overall, STs 5, 22, 36, 125, 228, 239, and 241 were found most often in cases of HA-MRSA, and STs 30 and 80 were found most often in cases of CA-MRSA. Strains acquired in North America or European regions were associated with HA-MRSA.

## Discussion

We have estimated risks for MRSA among travelers returning from specific geographic regions. Other studies have reported individual cases of MRSA diagnosed in connection with travel (*10*,*11*,*23*–*25*). Kaiser et al. (*12*) studied patients in the Netherlands who were repatriated after having received healthcare abroad, but found no association between country or region and MRSA diagnosis. However, the participation rate in their study was low, and the lack of statistically significant results could have been caused by the low number of cases.

The overall prevalence and transmission rate of MRSA within a country or region is just one of several factors that can influence the risk for MRSA acquisition by travelers to that country or region. This risk will also depend on type of exposure and duration of stay. For example, risk for MRSA acquisition will vary, depending on whether the person had received healthcare or had only community contacts, whether he or she stayed in a hotel at a holiday resort for a week, stayed with relatives for 3 months, or just stayed overnight on a business trip. In Europe, we observed a relatively high risk for MRSA acquisition in travelers to the United Kingdom, Ireland, and southern Europe, which is consistent with the high prevalence of MRSA reported for these regions (*26*–*28*). Other studies have shown that MRSA is hyperendemic to the Pacific region, East Asia, the Mediterranean region, and the Middle East (*27*–*29*). Thus, the higher risk for MRSA among travelers returning from these regions in our study may reflect such hyperendemicity. In addition, we found a higher risk for MRSA among travelers to sub-Saharan Africa and South America, and a risk for MRSA among travelers to central and eastern Europe that is comparable to that among travelers to southern Europe.

The risk for immigrants and adopted children of acquiring MRSA may not be representative of the overall risk in the general population in their respective countries of origin. These 2 populations may have spent time in institutional settings (e.g., orphanages, refugee camps, or hospitals) in which the risk for MRSA exposure may be different from that in the community in general. In addition, analysis of MRSA risk among newly adopted children and immigrants was based on small numbers of persons, which resulted in wide CIs of the estimated risks. Despite this uncertainty, agreement was seen between the estimated risk for MRSA acquisition among returning travelers, immigrants, and adopted children. Eight of 12 countries from which the adopted children with MRSA originated and 14 of the 22 countries from which immigrant cases originated belonged to the 6 regions with highest risk for MRSA among travelers (northeastern Mediterranean, South America, East Asia, Oceania and Pacific islands, sub-Saharan Africa, and North Africa and the Middle East).

Measuring the risk for MRSA in travelers, adopted children, and immigrants involved analyzing 3 different groups related to specified countries and regions. Because of the large degree of agreement between the 3 groups, results obtained may reflect not only risks for MRSA in these 3 populations, but also an overall comparatively higher risk for MRSA in some regions than in others.

The study was based on persons (carriers and those with clinical disease) with reported cases of MRSA in Sweden during 2000–2003. Only minor changes, which did not affect our primary findings, were seen when carrier cases were excluded from the travel analysis. Approximately 50% of MRSA cases among immigrants, but only 15% (6/40) of adopted children, had MRSA disease. Twelve of the 13 countries from which immigrants originated belonged to the 6 regions with highest risk for MRSA among travelers. For adopted children, the numbers of countries involved and cases decreased when carriers were excluded. However, 2 of 4 countries (4 of 6 cases) still belonged to the same 6 regions. Results for these 3 populations with acquired MRSA are consistent even when carriers were excluded from the analysis.

One strength of our study is that all regions/countries were investigated by using the same surveillance system, thus avoiding problems that might arise when data for different countries are compiled from different sources. Because the travel analysis was based on residents of Sweden who traveled to other countries and results were adjusted for sex and age, the main conclusions should also be valid for other western countries. Possible biases related to MRSA cases reported in Sweden during 2000–2003 and use of the TDB have been evaluated (*16*,*30*). Information from the TDB has shown good agreement with data from other sources (*31*). We could not control for length of stay of travelers at their destination because such information for MRSA cases was not available. However, persons with cases of MRSA who traveled for work purposes were commonly employed at the destination for a few months, rather than having been on short business trips.

Most persons infected with MRSA had HA-MRSA; only 33% had CA-MRSA. However, CA-MRSA showed a strong correlation with regions with a high risk for MRSA among travelers. This finding might be caused by a higher propensity among clinicians to sample patients with a history of activity (e.g., backpacking) under less than optimal sanitary conditions or travel to exotic regions. The 5 regions with the highest OR of acquiring MRSA in adjusted analysis were outside Europe.

Geographic distribution of MRSA STs was consistent with that in studies of other regions. ST22 and ST36 are well documented in the healthcare system in the United Kingdom (*32*). ST80 is common in the Mediterranean and Balkan region, the Middle East, and several countries in Europe, and epidemiologically related to persons with direct or indirect contact with the Mediterranean and Middle East (*1*,*11*,*12*,*23*–*25*). ST239 is common Asia (*33*,*34*) and the northeastern Mediterranean (*35*); ST30 is common in the Pacific region, East Asia, and Oceania (*1*,*36*); and ST228 is common in southern Europe and Germany (*37*,*38*).

In addition to showing an association between specific STs and regions, we also identified the transmission setting (healthcare or community) in which the isolate was acquired. We observed that most STs among imported cases of MRSA were associated with either HA-MRSA or CA-MRSA. ST30 and ST80 were strongly associated with CA-MRSA. ST72 was identified only among 8 adopted children from South Korea. However, information for the orphanages was not available. Thus, the possibility that 8 children could have been part of the same local transmission chain could not be ruled out. However, ST72 has recently been reported from several areas of South Korea among children and as a community-associated sequence type (*39*,*40*).

Several limitations of our study involve factors relevant to MRSA acquisition (length of stay abroad, contacts with institutions with a higher probability of MRSA transmission such as healthcare settings, orphanages, or refugee camps) that were not measured. In addition, there may have been inconsistencies in how MRSA cases were selected in the surveillance system. In some counties in Sweden, MRSA screening of adopted children is conducted; in other counties, it is not practiced. Thus, underdiagnosis of MRSA carriage among adopted children was likely. No similar screening recommendations are known for newly arrived refugees or immigrants.

In countries with a low prevalence of MRSA, a larger proportion of cases are imported than in countries with a high prevalence of MRSA. The risk for additional domestic transmission indicates a greater need to focus public health measures toward these imported cases. We observed differences in risk for MRSA among travelers returning from different regions, adopted children, and immigrants. Risk estimates for MRSA measured in these 3 groups showed a consistent pattern; some regions showed a higher risk for acquiring MRSA. Although most imported cases of MRSA were HA-MRSA, community acquisition showed a correlation with regions that have a high risk for MRSA among travelers. Knowledge of these differences in risk for MRSA will improve control measures and decrease domestic transmission.

## Supplementary Material

Appendix Table Cross-tabulation of region of acquisition and sequence type for cases of methicillin-resistant Staphylococcus aureus acquired abroad and reported in Sweden, 2000-2003*
