# Supplementary material for: Imported Methicillin-Resistant Staphylococcus aureus, Sweden
Source: Emerg Infect Dis. 2010 Feb;16(2):189–96. doi: 10.3201/eid1602.081655 (PMC2957988; doi:10.3201/eid1602.081655)
Supplement: Appendix Table — Cross-tabulation of region of acquisition and sequence type for cases of methicillin-resistant Staphylococcus aureus acquired abroad and reported in Sweden, 2000-2003* [file 08-1655_appT-s1.pdf]

Appendix Table. Cross-tabulation of region of acquisition and sequence type for cases of methicillin-resistant *Staphylococcus aureus* acquired abroad and reported in Sweden, 2000–2003*

ST	Sub-Saharan Africa	UK and Ireland	East Asia	Central and Eastern Europe	North Africa, Israel, Arabic countries, Iran	North America	Northeastern Mediterranean	Nordic	Oceania and Pacific islands	South America	Southern Europe	Western Europe	Total HA:CA (%, 95% CI)	Total
ST1														
HA			2	1									3 (27, 6–61)	11
CA			3	1									4 (36, 11–69)	
ST5														
HA	1		1			9				2		3	16 (67, 45–84)	24
CA					1	2				1			4 (17, 5–37)	
ST8														
HA	1	1	1	4		1			1			3	12 (39, 22–58)	31
CA		1	4	5		1				3			14 (45, 27–64)	
ST22														
HA		15	1					1	1		3		21 (88, 68–97)	24
CA	1	1											2 (8, 1–27)	
ST30														
HA			1		1		3						5 (18, 6–37)	28
CA			13			2	1		3				19 (68, 48–84)	
ST36														
HA	1	11	1			1	4				1	1	20 (95, 76–100)	21
CA													0	
ST45														
HA		1	1	1								3	6 (55, 23–83)	11
CA			1				1						2 (18, 2–52)	
ST72														
HA			2										2 (25, 3–65)	8
CA			6										6 (75, 35–97)	
ST80														
HA	1				2		1				1		5 (8, 3–19)	59
CA	2		2	2	18		21					1	46 (78, 48–84)	
ST88														

HA												1	1 (20, 0.5–72)	5
CA	3				1								4 (80, 28–99)	
ST125														
HA						1					5		6 (75, 35–97)	8
CA													0	
ST152														
HA							4						4 (67, 22–96)	6
CA							2						2 (33, 4–78)	
ST228														
HA				1			5				11	1	18 (95, 74–100)	19
CA													0	
ST239														
HA			22	1	5		30		3				61 (94, 85–98)	65
CA			1										1 (2, 0.04–8)	
ST241														
HA			4		5				1		1		11 (92, 62–100)	12
CA													0	
Total HA:CA (%, 95% CI)														
HA	7 (39, 17–64)	31 (94, 80–94)	43 (47, 36–57)	11 (46, 26–67)	20 (40, 26–55)	15 (71, 48–89)	56 (62, 51–72)	1	9 (53, 28–77)	5 (42, 15–72)	31 (86, 71–95)	15 (83, 59–96)	244 (59, 54–64)	
CA	9 (50, 26–74)	2 (6, 1–20)	37 (40, 30–51)	9 (38, 19–59)	20 (40, 26–55)	5 (21, 7–42)	29 (32, 23–43)	0	3 (21, 5–51)	4 (33, 10–65)	0	1 (6, 0.1–27)	119 (29, 24–33)	
Total	18	33	92	24	50	24	90	1	14	12	36	18		414

*Shown are 414 independent cases (not belonging to the same transmission chain) of 444 cases acquired abroad. Cases are stratified as healthcare-acquired (HA) and community-acquired (CA) for each sequence type (ST) and region of acquisition. In some instances, numbers in rows and columns do not add up to the totals because of persons with STs other than the ones listed or because cases were indeterminable with regard to transmission setting. CI, confidence interval.
